# Supplementary material for: The juxtamembrane linker of synaptotagmin 1 regulates Ca2+ binding via liquid-liquid phase separation
Source: Nat Commun. 2024 Jan 4;15:262. doi: 10.1038/s41467-023-44414-5 (PMC10766989; doi:10.1038/s41467-023-44414-5)
Supplement: Supplementary file 21 — Reporting Summary [file 41467_2023_44414_MOESM21_ESM.pdf]

nature portfolio

Corresponding author(s): Edwin Chapman

Last updated by author(s): Dec 7, 2023

Reporting Summary

Nature Portfolio wishes to improve the reproducibility of the work that we publish. This form provides structure for consistency and transparency in reporting. For further information on Nature Portfolio policies, see our Editorial Policies and the Editorial Policy Checklist.

Statistics

For all statistical analyses, confirm that the following items are present in the figure legend, table legend, main text, or Methods section.

n/a	Confirmed	
☐	☒	The exact sample size (*n*) for each experimental group/condition, given as a discrete number and unit of measurement
☐	☒	A statement on whether measurements were taken from distinct samples or whether the same sample was measured repeatedly
☐	☒	The statistical test(s) used AND whether they are one- or two-sided<br>*Only common tests should be described solely by name; describe more complex techniques in the Methods section.*
☒	☐	A description of all covariates tested
☒	☐	A description of any assumptions or corrections, such as tests of normality and adjustment for multiple comparisons
☐	☒	A full description of the statistical parameters including central tendency (e.g. means) or other basic estimates (e.g. regression coefficient) AND variation (e.g. standard deviation) or associated estimates of uncertainty (e.g. confidence intervals)
☐	☒	For null hypothesis testing, the test statistic (e.g. *F*, *t*, *r*) with confidence intervals, effect sizes, degrees of freedom and *P* value noted<br>*Give P values as exact values whenever suitable.*
☒	☐	For Bayesian analysis, information on the choice of priors and Markov chain Monte Carlo settings
☒	☐	For hierarchical and complex designs, identification of the appropriate level for tests and full reporting of outcomes
☒	☐	Estimates of effect sizes (e.g. Cohen's *d*, Pearson's *r*), indicating how they were calculated

Our web collection on statistics for biologists contains articles on many of the points above.

Software and code

Policy information about availability of computer code

Data collection	Data were acquired using:<br>Western blot: BioRad ChemiDoc MP v3.0.1.14<br>Fluorescence imaging: Zeiss 880 Airyscan LSM ZENv14.0.27.201, Zeiss Axiovert AX10 ZEN v3.5<br>Cartoon and schematics: Biorender, Adobe Illustrator 2023 v28.0<br>Isothermal titration calorimetry: MicroCal iTC200 v1.30, Origin 2020 v7.0552 (B552)<br>Protein sequence analysis: PONDR software (http://www.pondr.com/) vVL-XT<br>Dynamic light scattering: Wyatt NanoStar2 v1.1.2.3<br>Simulations: GROMACS 2020.1 software package<br>Protein structure visualization: AlphaFold
Data analysis	Contact pair analysis: PLUMED 2.5.3<br>Trajectory viewing: VMD 1.9.3<br>Graphing and plotting: Graphpad PRISM 9.2<br>Image analysis: Fiji v1.54g<br>Sequence alignment: UniProt and ClustalOmega (https://www.ebi.ac.uk/Tools/msa/clustalo/)<br>Data analysis: Excel 2019

For manuscripts utilizing custom algorithms or software that are central to the research but not yet described in published literature, software must be made available to editors and reviewers. We strongly encourage code deposition in a community repository (e.g. GitHub). See the Nature Portfolio guidelines for submitting code & software for further information.

Data

Policy information about availability of data

All manuscripts must include a data availability statement. This statement should provide the following information, where applicable:

- Accession codes, unique identifiers, or web links for publicly available datasets
- A description of any restrictions on data availability
- For clinical datasets or third party data, please ensure that the statement adheres to our policy

Source data for the relevant figures and tables are provided as a Source Data file. Simulation parameters, GROMACS input files, and MD simulation final coordinate files are provided as Supplementary Dataset. Uniprot (syt1 accession IDs: P21707 (Organism: Rattus norvegicus), P46096 (Organism: Mus musculus), P21579 (Organism: Homo sapiens), P47191 (Organism: Gallus gallus), P48018 (Organism: Bos taurus), P21521 (Organism: Drosophila melanogaster)) and Research Collaboratory for Structural Bioinformatics (RCSB) Protein Data Bank (PDB) (syt1 PDB IDs: 1RSY, 1K5W) databases were used for sequence and structure analysis.

Research involving human participants, their data, or biological material

Policy information about studies with human participants or human data. See also policy information about sex, gender (identity/presentation), and sexual orientation and race, ethnicity and racism.

Reporting on sex and gender	Not required for our study
Reporting on race, ethnicity, or other socially relevant groupings	Not required for our study
Population characteristics	Not required for our study
Recruitment	Not required for our study
Ethics oversight	Not required for our study

Note that full information on the approval of the study protocol must also be provided in the manuscript.

Field-specific reporting

Please select the one below that is the best fit for your research. If you are not sure, read the appropriate sections before making your selection.

[x] Life sciences [ ] Behavioural & social sciences [ ] Ecological, evolutionary & environmental sciences

For a reference copy of the document with all sections, see nature.com/documents/nr-reporting-summary-flat.pdf

Life sciences study design

All studies must disclose on these points even when the disclosure is negative.

Sample size	Sample sizes were not predetermined using statistical methods; instead, they were determined by considering the variability of results in each experiment. The selected sample sizes for each experiment were adequate to reveal consistent trends in the data for analysis.
Data exclusions	No data were excluded during the analysis.
Replication	All experiments were repeated independently at least thrice on separate days with unique materials, as specified in the figure legends. Replicates were successfully reproducible.
Randomization	No formal randomization was performed and was not relevant to our study. During acquisition, each dataset was acquired randomly and discontinuously that controlled for systematic error. All samples and dataset were included in the analysis.
Blinding	For data analysis, all the datasets (ITC experiments, MD simulation contact analysis, and fluorescence images) were de-identifed and then processed.

Reporting for specific materials, systems and methods

We require information from authors about some types of materials, experimental systems and methods used in many studies. Here, indicate whether each material, system or method listed is relevant to your study. If you are not sure if a list item applies to your research, read the appropriate section before selecting a response.

Materials & experimental systems

n/a	Involved in the study	
	x	Antibodies
	x	Eukaryotic cell lines
x		Palaeontology and archaeology
	x	Animals and other organisms
x		Clinical data
x		Dual use research of concern
x		Plants

Methods

n/a	Involved in the study	
x		ChIP-seq
x		Flow cytometry
x		MRI-based neuroimaging

Antibodies

Antibodies used

anti-GFP [Roche, 11814460001, clones 7.1 and 13.1, Lot 47859600]
anti-syt1 [Developmental Studies Hybridoma Bank, mAb48, clone asv 48]
anti-β-actin [Cell Signaling Technology, 3700, clone 8H10D10, Lot 21]
Goat anti-Mouse IgG-HRP [Bio-Rad Laboratories, 1706516, RRID:AB_11125547, Lot 64449023]
anti-syt1 [Synaptic Systems, 105 103]
anti-syp [Synaptic Systems, 101 004, Lot 3-38]
goat anti-rabbit IgG Alexa Fluor 594 [Thermo Fisher Scientific, A11037, Lot 1777945]
goat anti-guinea pig IgG Alexa Fluor 647 [Thermo Fisher Scientific, A21450, Lot 2446026]

Validation

All the antibodies used in this paper are commercially available and tested by manufacturers with detailed specificity described on their websites. Our lab have used these antibodies on a regular basis.

anti-GFP [Roche, 11814460001]: Suitable for WB, ICC; Reacts with rat , mouse.
https://www.sigmaaldrich.com/US/en/product/roche/11814460001

anti-syt1 [Developmental Studies Hybridoma Bank, mAb48]: Suitable for WB, ICC; Reacts with human, rat , mouse.
https://dshb.biology.uiowa.edu/mAb-48-asv-48

anti-β-actin [Cell Signaling Technology, 8H10D10]: Suitable for WB, ICC; Reacts with human, rat , mouse.
https://www.cellsignal.com/products/primary-antibodies/b-actin-8h10d10-mouse-mab/3700

Goat anti-Mouse IgG-HRP [Bio-Rad Laboratories, 1706516]: Suitable for WB; Reacts with mouse.
https://www.bio-rad.com/en-us/sku/1706516-goat-anti-mouse-igg-h-l-hrp-conjugate?ID=1706516

anti-syt1 [Synaptic Systems, 105 103]: Suitable for WB, ICC; Reacts with rat , mouse.
https://sysy.com/product/105103

anti-syp [Synaptic Systems, 101 004]: Suitable for WB, ICC; Reacts with human, rat , mouse.
https://sysy.com/product/101004#list

goat anti-rabbit IgG Alexa Fluor 594 [Thermo Fisher Scientific, A11037]: Suitable for ICC; Reacts with rabbit.
https://www.thermofisher.com/antibody/product/Goat-anti-Rabbit-IgG-H-L-Highly-Cross-Adsorbed-Secondary-Antibody-Polyclonal/A-11037

goat anti-guinea pig IgG Alexa Fluor 647 [Thermo Fisher Scientific, A21450]: Suitable for ICC; Reacts with guinea pig.
https://www.thermofisher.com/antibody/product/Goat-anti-Guinea-Pig-IgG-H-L-Highly-Cross-Adsorbed-Secondary-Antibody-Polyclonal/A-21450

Eukaryotic cell lines

Policy information about cell lines and Sex and Gender in Research

Cell line source(s)	HEK-293T [ATCC, CRL-3216]
Authentication	HEK-293T cell line has been commonly used in our lab. It was further authenticated by observing its morphology.
Mycoplasma contamination	Cell lines were free of any contamination
Commonly misidentified lines (See ICLAC register)	No commonly misidentified cell lines were used in this study.

Animals and other research organisms

Policy information about studies involving animals; ARRIVE guidelines recommended for reporting animal research, and Sex and Gender in Research

Laboratory animals	Hippocampal neurons were dissected from pre-natal Sprague-Dawley rats on E18 (Envigo) on P0-P1. (https://www.inotivco.com/model/hsd-sprague-dawley-sd). Rats were housed in an environment of suitable temperature (25 °C) and humidity (typically 50%) under a 12 h light-dark cycle.
Wild animals	The study did not involve any wild animals.
Reporting on sex	Sex was not considered in this study design and methods.
Field-collected samples	The study did not involve any field-collected samples.
Ethics oversight	Animal care and use in this study were conducted under guidelines set by the National Institutes of Health's Guide for the care and use of laboratory animals handbook. Protocols were reviewed and approved by the Animal Care and Use Committee at the University of Wisconsin–Madison (Laboratory Animal Welfare Public Health Service Assurance Number: A3368-01)

Note that full information on the approval of the study protocol must also be provided in the manuscript.
